# Commonalities in frailty and psychopathology predict chronotype across severe mental disorders from a comorbidity perspective

**DOI:** 10.1017/S0033291726104425

**Published:** 2026-05-12

**Authors:** Joan Vicent Sánchez-Ortí, Patricia Correa-Ghisays, Vicent Balanzá-Martínez, Gabriel Selva-Vera, Victor M. Victor, Constanza San Martin Valenzuela, Pau Soldevila-Matías, María Flores-Rodero, Jon Sánchez-Valle, Jaume Forés-Martos, Diego Macías Saint-Gerons, Inmaculada Fuentes-Durá, Alfonso Valencia, Rafael Tabarés-Seisdedos

**Affiliations:** 1 INCLIVA - Health Research Institute, Valencia, Spain; 2 TMAP - Evaluation Unit in Personal Autonomy, Dependency and Serious Mental Disorders, University of Valencia, Valencia, Spain; 3 Center for Biomedical Research in Mental Health Network (CIBERSAM), Health Institute, Carlos III, Madrid, Spain; 4 Faculty of Psychology, University of Valencia, Valencia, Spain; 5 Teaching Unit of Psychiatry and Psychological Medicine, Department of Medicine, University of Valencia, Valencia, Spain; 6 VALSME (VALencia Salut Mental i Estigma) Research Group, University of Valencia, Valencia, Spain; 7 Department of Physiology, Faculty of Medicine and Dentistry, University of Valencia, Valencia, Spain; 8 Service of Endocrinology and Nutrition, University Hospital Dr. Peset, Valencia, Spain; 9 Foundation for the Promotion of Health and Biomedical Research in the Valencian Region (FISABIO), Valencia, Spain; 10 Department of Physiotherapy, University of Valencia, Valencia, Spain; 11 Department of Psychology, Faculty of Health Sciences, European University of Valencia, Valencia, Spain; 12 Computational Biology Group, Life Sciences Department, Barcelona Supercomputing Center, Barcelona, Spain; 13 Faculty of Nursery, University of Valladolid, Valladolid, Spain; 14 Catalan Institution for Research and Advanced Studies (ICREA), Barcelona, Spain

**Keywords:** comorbidity, evening chronotype, frailty, functional performance, hematological biomarkers, neurocognitive

## Abstract

**Background:**

Individuals with severe mental illness (SMI) have increased risk of physical comorbidities, linked to worse outcomes such as greater psychopathology, frailty, and neurocognitive impairment. Mechanisms underlying this burden remain unclear. This study examined whether frailty and psychopathology predict evening chronotype, especially in SMI with comorbidities.

**Methods:**

A longitudinal study assessed 165 participants at two time points over one year: schizophrenia (*n* = 30), bipolar disorder (*n* = 42), major depressive disorder (*n* = 35), and healthy controls (*n* = 58). The SMI group (*n* = 107) was divided into SMI with comorbidities (SMI-C; *n* = 47) and without (SMI; *n* = 60). Measures included psychopathology, frailty, chronotype, neurocognitive and functional performance, and hematological biomarkers.

**Results:**

Neurocognitive and functional impairments were greater in SMI groups than controls (*F* = 10.3–31.4; *p* < 0.0001; η²*p* = 0.12–0.34). The SMI-C group showed worse frailty than controls at T1 (*F* = 4.3; *p* < 0.01; η²*p* = 0.05) and than SMI at T2 (*F* = 8.5; *p* < 0.0001; η²*p* = 0.12), and elevated MCV/MCH (*F* = 3.8–9.4; *p* < 0.05–0.0001; η²*p* = 0.04–0.11). Chronotype distribution did not differ. Frailty and psychopathology predicted chronotype in SMI (*p* < 0.05–0.01); in controls, frailty and performance did so (p < 0.05).

**Conclusions:**

Psychopathological and hematological profiles are associated with chronotype and may help identify subgroups for chronobiology-informed interventions. These findings support more personalized treatment approaches.

## Highlights

- Severe mental illness (SMI) is associated with increased physical comorbidities, frailty, neurocognitive impairment, and greater psychopathology.
- Patients with SMI and comorbidities (SMI-C) showed poorer functional outcomes and elevated hematological markers (MCV, MCH).
- Frailty, neurocognitive performance, and psychopathology prospectively predicted evening chronotype across groups.
- Combined psychopathological and hematological profiles best discriminated evening chronotype, highlighting chronobiological therapeutic targets.

## Introduction

Severe mental illnesses (SMIs), including major depressive disorder (MDD), bipolar disorder (BD), and schizophrenia-spectrum disorders (SZ), are among the leading contributors to global disease burden and are frequently accompanied by multiple physical comorbidities (GBD, 2020, 2022; Nordgaard, Nielsen, Rasmussen, & Henriksen, 2023). For instance, it has been observed that they experience higher rates of metabolic syndrome, type II diabetes mellitus (T2DM), myocardial infarction, stroke, cancer, and neurodegenerative disorders (Halstead et al., 2024). Moreover, these physical conditions are frequently underdiagnosed and undertreated in individuals with SMIs, contributing to increased morbidity and mortality (Moeti, Gao, & Herrman, 2022; Peitz et al., 2021).

Comorbidity, the co-occurrence of multiple diseases in the same individual, is associated with poorer quality of life and impaired social and cognitive functioning (GBD, 2024). In terms of neurocognitive functioning, specific comorbidity profiles, particularly cardiometabolic comorbidities, traumatic brain injuries, and SMIs, have been associated with an increased likelihood of developing dementia (Menegon et al., 2024). Moreover, mood and psychotic symptoms are significantly associated with greater cognitive decline in individuals with existing physical diseases (Arnaud et al., 2022; Hagi et al., 2021; Kang et al., 2024).

A higher number of comorbidities is associated with lower functional performance in individuals with neurocognitive impairment (Temedda, Garnier-Crussard, Mouchoux, & Dauphinot, 2024). This relationship has also been observed in individuals with SMIs (Castro-de-Araujo et al., 2022; Rodrigues et al., 2021). Comorbidities are believed to contribute significantly to the development of frailty. Frailty, which is defined as a decline in functioning across multiple physiological systems accompanied by increased vulnerability to stressors, is a common risk factor for physical and mental illnesses (Hoogendijk et al., 2019). For instance, frail individuals with mood or psychotic disorders exhibit lower neurocognitive and functional performance compared to both nonfrail individuals and the general population (Pearson et al., 2022).

It should be noted that frailty has also been linked to disruptions in circadian regulation, suggesting that physiological vulnerability across multiple systems may interact with circadian preferences and behavioral rhythms. Chronotype refers to individual differences in the preferred time of day for performing daily activities and neurocognitive performance. Extreme chronotype preferences, particularly the evening type, have been associated with poorer physical health, metabolic dysregulation, and an increased risk of mental disorders (Zou et al., 2022). Circadian rhythms regulate multiple physiological processes, including immune and hematological activity, suggesting that chronotype may reflect broader systemic biological states.

Extreme chronotype preferences, particularly the evening chronotype, have been associated with poorer physical health, metabolic dysregulation, and an increased risk of mental disorders. Recent studies indicate that the evening chronotype is linked to schizophrenia, antidepressant resistance in MDD, and somatic comorbidities (Hebl, Velasco, & McHill, 2022; Krupa, 2025; Tsapakis, Fountoulakis, Kanioura, & Einat, 2024). This evidence highlights the relevance of chronotype as a potential marker of systemic vulnerability in individuals with SMI.

An increasing amount of research is investigating chronotype as a predictor of clinical and physical health outcomes. The evening chronotype, in particular, has been linked to a higher risk of cardiometabolic diseases, reduced emotional well-being, and worse psychiatric outcomes. These insights imply that circadian preference might serve as a simple, observable indicator to identify individuals at increased risk for negative health trajectories and to inform preventive approaches in clinical settings (Hebl et al., 2022; Tsapakis et al., 2024). However, few studies have examined the relationship between chronotype and comorbidities in individuals with SMI. A recent study showed that individuals at high clinical risk for psychosis and mood disorders have a greater preference for the evening chronotype than the general population. Notably, mental comorbidities were not associated with chronotype preference. No data were reported regarding physical comorbidities (Lunsford-Avery et al., 2021).

Therefore, far fewer studies have investigated this relationship from the opposite perspective: whether the clinical and biological features commonly seen in severe mental illnesses might themselves influence chronotype. Identifying which psychopathological, functional, or biological traits are linked to circadian preference could help uncover transdiagnostic vulnerability profiles and clarify whether chronotype indicates a broader systemic dysregulation, rather than functioning solely as a precursor trait.

Changes in red blood cell (RBC) parameters have been associated with various physical comorbidities in patients with SMI (Sánchez-Valle et al., 2020; Teixeira, Martins, Berk, & Bauer, 2022). Beyond their role in oxygen transport, erythrocytes are increasingly recognized as markers of systemic metabolic and inflammatory processes, which in turn are subject to circadian regulation. Therefore, variations in hematological parameters, such as red blood cell count, mean corpuscular volume (MCV), and mean corpuscular hemoglobin (MCH), may reflect circadian rhythm-related physiological alterations linked to cardiometabolic and neuropsychiatric vulnerability.

Likewise, there is also growing evidence implicating RBC changes in neurocognitive and functional impairments, as well as increased frailty in individuals with cardiometabolic diseases (e.g. metabolic syndrome and hypertension) and central nervous system disorders (e.g. Alzheimer’s disease and amyotrophic lateral sclerosis) (Prudinnik et al., 2025). In this context, elevated hematological activity, such as RBC, MCV, and MCH, associated with evening chronotypes has been observed in some cardiometabolic diseases such as hypertension, diabetes, and obesity (Baldanzi et al., 2022). However, this relationship has not been explored in populations with SMIs.

To the best of our knowledge, no studies have examined the relationship between chronotype and health status in individuals with SMI from a combined transdiagnostic and comorbidity perspective. This research gap hinders a better understanding of whether clinical outcomes (such as comorbidities, frailty, and cognitive and functional performance) and hematological biomarkers (such as RBCs) can help identify individuals with evening chronotypes. We hypothesized that frailty and psychopathology would predict evening chronotype, particularly in SMI with physical comorbidities.

## Materials and methods

### Study design and ethical considerations

This study was part of a larger project aimed at identifying and validating peripheral biomarkers of neurocognitive deficits in patients with major depressive disorder (MDD), bipolar disorder (BD), schizophrenia-spectrum disorder (SZ), and type 2 diabetes mellitus (T2DM). This prospective, comparative cohort study was conducted between April 2015 and January 2018 to investigate the association and evolution of specific peripheral blood biomarkers of neurocognitive impairment in a unique longitudinal cohort of individuals with physical diseases and SMIs. Demographic and clinical data, neurocognitive and functional data, and information on peripheral blood biomarkers were collected at baseline (T1) and after 1 year (T2). Individuals with SMIs were recruited from mental health units (MHUs) in several towns in the province of Valencia, Spain (Gandía, Foios, Catarroja, Paterna, and Sagunto), the psychiatry outpatient clinic, the endocrinology department of the University Hospital, Dr. Peset, and Miguel Servet MHU in Valencia City. Healthy controls (HCs) were residents from the same geographical area and were demographically matched to the SMI group. The participants were demographically matched. Participants were recruited by convenience sampling and were not a random sample representative of the local population. Additional details on participant selection and recruitment procedures have been described previously (Correa-Ghisays et al., 2022). Written informed consent was obtained from all participants after the study procedure was fully explained. The study protocol was approved by the ethics committee and the institutional review board of each participating center, and the ethical principles of the Declaration of Helsinki were followed. Only the variables related to the objectives of the present study were included in the analyses.

### Participants

MDD, BD, and SZ were diagnosed according to the criteria in the Diagnostic and Statistical Manual of Mental Disorders 5 (DSM-5) (Association, 2014). Participants with MDD or BD were required to meet remission criteria for an acute affective episode (Tohen et al., 2009). Individuals with SZ also meet the remission criteria for psychotic episodes (Andreasen et al., 2005). To recruit HCs, the absence of pharmacological treatment and a family history of psychiatric disorders in first-degree relatives were required. The ability to understand the study procedures and willingness to provide written consent were required for participation. General exclusion criteria for all groups included current hospitalization; documented cognitive impairment not secondary to psychiatric disorders, such as intellectual disability or significant neurocognitive disorders, that is, dementia; any condition impairing comprehension of the protocol; current substance use disorders (except nicotine); pregnancy; intake of steroids, corticosteroids, antioxidants, antibiotics, and immunologic therapies; fever with temperature over 38 °C; and history of vaccination within 4 weeks of evaluation. The same inclusion and exclusion criteria were systematically applied to all participants in these analyses, based on the presence of a severe mental disorder and comorbidities. No separate analysis was conducted for T2DM.

The exposure of interest was the presence of one or more physical comorbidities measured using the Charlson Comorbidity Index (CCI) questionnaire (Rius et al., 2004). Comorbidities were examined at both time points. Given the known limitations of the CCI (Drosdowsky & Gough, 2022), it was verified that the number (i.e. multicomorbidity status) and severity of comorbid conditions were similarly distributed among the individuals with SMIs (MDD, BD, and SZ) (Charlson, Carrozzino, Guidi, & Patierno, 2022) (see Supplementary Material 1). Accordingly, participants with a CCI score ≥ 1 were classified as SMI together with physical comorbidity (SMI-C), and those with a CCI score = 0 were classified as SMI. The remaining participants were classified as HCs.

### Clinical and neuropsychological assessment

All assessments were conducted by the same experienced psychologists and psychiatrists from the research group. Sociodemographic data, including sex, age, years of education, and intelligence quotient (IQ) estimated using the vocabulary subtest of the Wechsler Adult Intelligence Scale, Third Edition (WAIS-III) (Krull, Scott, & Sherer, 1995), dependent status, and motor laterality (manual, ocular, and crural dominance) were collected. Psychopathology severity was assessed using the Clinical Global Impression (CGI) Scale (Haro et al., 2003; Vieta et al., 2002). Ages at onset and illness duration were also recorded.

Clinical evaluations were conducted using the following instruments: (i) 17-item Hamilton Rating Scale for Depression (HDRS) (Ramos-Brieva & Cordero-Villafáfila, 1986), (ii) Young Mania Rating Scale (Colom et al., 2002), and (iii) Positive and Negative Syndrome Scale (PANSS) (Peralta & Cuesta, 1994).

Neurocognitive performance was assessed using a comprehensive battery of neuropsychological tests and subtests previously applied by our group (GIUV2016–312, CB/07/09/0021; see Correa-Ghisays et al., 2022, for the full description of the neurocognitive battery). The battery included seven cognitive domains: (i) learning and verbal memory (L&VM): Complutense Verbal Learning Test (TAVEC) for total immediate recall, short-term free recall, and long-term free recall variables (Benedet & Alejandre, 2014); (ii) cognitive flexibility (CF): Stroop Color and Word Test (SCWT) color/word subtest (Golden, 2001) and Wisconsin Card Sorting Test categories for completed and perseverative errors (Grant & Berg, 2001); (iii) verbal fluency (VF): FAS and animal-naming test for phonemic and semantic fluency, respectively (Reitan & Wolfson, 1985); (iv) working memory (WM): Trail-Making Test (TMT) Part B (Reitan & Wolfson, 1985) and WAIS-III digit span backward (Wechsler, 1999); (v) short-term memory (StM): TAVEC immediate recall of the first learning trial, immediate recall of the interference list (Benedet & Alejandre, 2014), and WAIS-III digit span forward (Wechsler, 1999); (vi) visual memory (VM): Rey-Osterrieth Complex Figure Test (ROCFT) 2 min after the copy (fRey2) and 20 min after the copy (fRey20) (Rey, 1999); and (vii) processing speed (PS): Finger-tapping test (left unimanual, right unimanual, left bimanual, right bimanual, and average of the four scores) (Reitan & Wolfson, 1985; Tabarés-Seisdedos et al., 2003), WAIS-III digit symbol coding subtest (Wechsler, 1999), SCWT color and word subtests (Golden, 2001), and TMT Part A (Reitan & Wolfson, 1985). A global cognitive score (GCS) was calculated by averaging the scores across the seven cognitive domains.

Functional performance was evaluated using the Functional Assessment Short Test (FAST) (Rosa et al., 2007), Short Form-36 Health Survey questionnaire (SF-36) (Alonso, Prieto, & Antó, 1995), and World Health Organization Quality of Life Brief Scale (WHO-QoL-BREF) (Bobes et al., 2004). The global functional score (GFS) was calculated by averaging the total scores of the three scales.

We standardized and normalized the scores obtained from the questionnaires used to assess neurocognitive and functional performance, taking the maximum scores of each questionnaire and the values obtained by the HC group as a reference. Cluster analysis was performed to confirm that the individual questionnaire scores could be grouped into two global constructs, which we refer to as GCS and GFS. We also inverted the questionnaire scores in cases where it was necessary to perform the calculation correctly (Correa-Ghisays et al., 2022).

Frailty performance was measured using an electronic dynamometer (NedVEP/IBV) with a built-in extensometric transducer and the NedDiscapacidad/IBV software V4.1.1 from the Valencia Institute of Biomechanics (see Supplementary Material 2). Each dynamometer was calibrated for each participant before each test. The test was performed with the participant sitting in an upright position on a chair with and without a backrest. Their feet were supported on the floor with 90° knee flexion. The arm was positioned with 90° elbow flexion and neutral pronosupination of the forearm. Hand strength was measured in three functional positions: handgrip, lateral/key pinch (thumb pad and lateral aspect of the index finger), and tip pinch (thumb opposed by the index and long fingers), as previously described. For each functional position, three maximum strength scores (*N* or kg) were obtained for both hands. If repetitions within a hand varied by <10%, the values were averaged per hand (El Assar, Rodríguez-Sánchez, Álvarez-Bustos, & Rodríguez-Mañas, 2024). The global frailty score (GFRS) was calculated by averaging the total scores of the hand strength measures for different functional positions.

Chronotype was assessed using the reduced version of the Morningness-Eveningness Questionnaire (Eveningness) (Adan & Almirall, 1989, 1991). The chronotypes were examined continuously and categorically.

This study used the validated Spanish version of all clinical, cognitive, functional, frailty, and chronotype assessment instruments.

### Determination of hematological biomarkers in blood

Venous blood extraction was performed, and serum and plasma samples were stored in a freezer at −80 °C. A lysis buffer was added to the residual erythrocytes in the pellet, which was then incubated at room temperature for 5 min and centrifuged at 240 × g for 5 min.

The following blood count parameters were considered: RBC count, hemoglobin (HGB), hematocrit (HCT), mean corpuscular volume (MCV), mean corpuscular hemoglobin (MCH), mean corpuscular hemoglobin concentration (MCHC), and red blood cell distribution width (RDW).

### Statistical analyses

Data were analyzed using the Statistical Package for Social Sciences (version 26.0; IBM Corp., 2019). Sample size estimation was conducted using Ene 2.0 software, based on the ability to detect a moderate effect size (Cohen’s *d* = 0.5) on the primary outcome variable (overall cognitive score) with 80% power and *α* = 0.05. This calculation showed that a minimum of 29 participants per group was necessary (Pedromingo-Marino, 2005).

Descriptive analyses were performed using one-way analysis of variance (ANOVA) Student’s *t*-test for continuous variables, and the chi-square test for categorical variables. Group differences in psychiatric symptoms, neurocognitive and functional performance, frailty, chronotype, and hematological biomarkers at T1 and T2, as well as changes over time, were assessed using mixed multivariate analysis of covariance (MANCOVA) with age and years of education as covariates. Normality was assumed for all continuous variables based on the Kolmogorov–Smirnov test, which confirmed the appropriateness of parametric MANOVA/MANCOVA tests at both time points. A post-hoc analysis with Bonferroni-corrected pairwise *t*-tests and Mann–Whitney *U*-tests was performed to examine group differences. Effect sizes for between-group comparisons were expressed as partial eta-squared (*η* ^2^*p*), using the conventional thresholds: small (*η* ^2^*p* = 0.01), medium (*η* ^2^*p* = 0.06), and large (*η* ^2^*p* = 0.14), in accordance with Cohen (1988).

Dropout analyses revealed no significant differences between participants who completed follow-up and those who were lost to follow-up in terms of age, sex, diagnosis, or initial clinical severity (all *p* > 0.05). Raw scores for neurocognitive and functional performances and frailty were transformed into *z*-scores. The mean and standard deviation of the HCs at T1 were used as reference values to calculate the *z*-scores. All *z*-scores were standardized so that higher values consistently indicated poorer performance.

Linear regression analysis was used to evaluate the predictive value of psychiatric symptoms, neurocognitive and functional performance, frailty, and hematological biomarkers at baseline (T1) in explaining variance in chronotype at T2. Multicollinearity was assessed using the variance inflation factor (VIF). All predictor variables had VIF values below 2.0, indicating that there is no significant multicollinearity. Other relevant variables (e.g. age) related to chronotype were not included because they were not the focus of this study, and clinical outcomes and hematological biomarkers were considered optimal predictors. Statistical significance was set at *p* < 0.05. Age and years of education were not included in the final regression model due to the lack of a significant association in the preliminary analyses and to avoid overfitting the model, given the sample size. The predictive modeling followed a stepwise procedure. First, linear regression analyses were conducted separately for each biomarker, and predictive models were generated that included statistically significant variables (*p* < 0.05). Finally, the optimal predictive combination was obtained. Missing data were minimal (<5%) and were handled using listwise deletion in the regression models.

Similarly, to test the predictive ability of psychiatric symptoms, neurocognitive and functional performance, frailty, and hematological biomarkers at T1 to discriminate individuals with evening chronotypes over time, binary logistic regression was performed. Predictive models were constructed for each group (based on comorbidity status) using only the variables that were statistically significant.

## Results

### Sample description

At T1, the sample consisted of 165 individuals: 35 with MDD, 42 with BD, 30 with SZ, and 58 HCs. Participants with SMIs (*n* = 107) were stratified into two groups based on the presence of physical comorbidity at both time points, comprising 60 in the SMI group (MDD = 18, BD = 22, and SZ = 20) and 47 in the SMI-C group (MDD = 17, BD = 20, and SZ = 10).

Forty participants were lost to follow-up at T2 (MDD = 25, BD = 29, and SZ = 27; HC = 44) (retention rate, 75.7%). At T2, the sample comprised 48 individuals in the SMI group (MDD = 10, BD = 19, and SZ = 19) and 33 individuals in the SMI-C group (MDD = 15, BD = 10, and SZ = 8).

Baseline sociodemographic and clinical characteristics of the SMI, SMI-C, and HCs groups are summarized in Table 1. Females accounted for 48% of the participants. The mean age of the total sample was 46.2 (SD: 12.3) years. The HC group had a significantly higher number of years of education. Patients in the SMI group were significantly younger than those in the SMI-C group. Age at onset, illness duration, and CGI were similar between the SMI groups.

**Table 1.** Sociodemographic characteristics of the sample at T1

Variables^a^	HC	SMI	SMI-C	Statistical analyses	
	(*n* = 58)	*(n* = 60)	(*n* = 47)	*F*(*p*)	Post hoc test
*Sociodemographic*					
Sex^b^ ^,^^c^	27 (47%)	29 (48%)	24 (51%)	NS	
Age^d^ ^,^^e^	47.3 (15.9)	43.4 (11.3)	50.5 (9.9)	4.1**	SMI < SMI-C
Years of education^d^ ^,^^e^	14.3 (5.1)	11.9 (4.0)	10.7 (4.2)	8.5****	SMI-C,SMI < HC
IQ^d^	113.3 (13.5)	112.8 (17.2)	108.2 (17.9)	NS	
Dependent status^c^ ^,^^f^	10 (17%)	19 (32%)	12 (26%)	NS	
Laterality^c^ ^,^^g^	50 (86%)	55 (91%)	45 (96%)	NS	
*Clinical*					
Age of onset^h^ ^,^^i^	–	28.4 (9.0)	29.8 (12.0)	NS	
Illness duration^h^ ^,^^i^	–	17.6 (7.7)	21.2 (12.2)	NS	
CGI^i^ ^,^^j^	–	3.8 (1.2)	3.7 (1.1)	NS	

*Note*: HC, healthy control; SMI, severe mental illness; C, comorbidity; IQ, intelligence quotient; CGI, clinical global impression; NS, not significant.

a Expressed as mean (standard deviation), except when indicated.

b Female *n* (%).

c Chi-squared test.

d ANOVA.

e Bonferroni test.

f Dependent *n* (%).

g Right-handers *n* (%).

h Years.

i *t*-test for independent samples.

j Lower scores represent a better outcome.

(NS = *p* > 0.05; *p ≤ 0.05; **p ≤ 0.01; ***p ≤ 0.001; ****p ≤ 0.0001).

### Analysis of loss to follow-up

Given the longitudinal design, we examined whether participants who remained in the study at T2 differed from those who were lost to follow-up. No significant differences were observed between participants who remained in the study and those who dropped out in terms of age, sex, distribution of diagnoses, psychiatric symptoms, neurocognitive performance, functional status, frailty, or hematological parameters at T1 (all *p* > 0.05). These findings suggest that dropout was unlikely to introduce systematic bias into the longitudinal analyses.

### Between-group comparison of psychiatric symptoms, neurocognition, functional performance, frailty, and chronotype outcomes

Psychiatric symptoms, neurocognitive and functional performance, frailty, and chronotype outcomes for all groups at T1 and T2 are presented in Table 2. Depressive symptoms were significantly higher in both SMI and SMI-C groups compared to the HC group at T1 (*p* < 0.0001; *η* ^2^*p* = 0.15), and at T2, the SMI-C group showed significantly higher depressive symptoms compared to the SMI group (*p* < 0.0001; *η* ^2^*p* = 0.23), indicating a gradient effect associated with physical comorbidity. Furthermore, individuals with SMIs exhibited significantly higher psychotic symptoms than the HCs at both times (*p* < 0.01–0.0001; *η* ^2^*p* = 0.05–0.21). Notably, no differences in manic symptoms were observed between the groups at either time point.

**Table 2.** Psychiatric symptoms, neurocognition, functional performance, frailty, and chronotype outcomes. Between-group comparison at T1 and T2

Variables^a^	HC		SMI		SMI-C		Statistical analyses					
	T1 (*n* = 58)	T2 (*n* = 44)	T1 *(n* = 60)	T2 (*n* = 48)	T1 (*n* = 47)	T2 (*n* = 33)	T1 F(*p*)^b^	Post hoc test^c^	*η* ^2^*p* ^d^	T2 F(*p*)^b^	Post hoc test^c^	*η* ^2^*p* ^g^
*Psychiatric symptoms*												
HDRS^e^	3.0 (3.2)	2.5 (4.3)	7.5 (6.2)	6.6 (6.1)	9.4 (7.1)	12.0 (8.7)	14.2****	HC < SMI,SMI-C	.15	17.9****	HC < SMI,SMI-C SMI < SMI-C	.23
YMRS^e^	1.2 (1.9)	0.6 (1.0)	2.8 (4.4)	2.2 (3.9)	3.0 (4.0)	1.7 (3.1)	NS			NS		
PANSS-P^e^	7.0 (0.0)	7.0 (0.0)	9.0 (4.3)	8.5 (3.1)	8.1 (2.3)	8.4 (3.1)	4.3**	HC < SMI	.05	NS		
PANSS-N^e^	7.0 (0.5)	7.0 (0.5)	13.1 (9.8)	9.3 (3.9)	10.8 (5.8)	10.2 (6.4)	8.3****	HC < SMI	.09	4.5**	HC < SMI-C	.07
PANSS-G^e^	16.5 (1.7)	16.2 (1.3)	24.3 (12.0)	20.2 (5.6)	24.5 (10.5)	24.2 (8.8)	8.4****	HC < SMI,SMI-C	.09	15.9****	HC < SMI,SMI-C SMI < SMI-C	.21
*Neurocognition, functional performance, and frailty*												
GCS^f^	0.0 (1.0)	0.0 (0.9)	−0.7 (1.0)	−0.5 (0.9)	−1.1 (0.9)	−1.1 (1.0)	11.0****	HC > SMI,SMI-C	.12	10.3****	HC > SMI,SMI-C	.14
GFS^f^	0.0 (1.0)	0.0 (0.9)	−1.0 (1.1)	−0.9 (1.1)	−1.7 (1.2)	−2.1 (1.3)	25.4****	HC > SMI,SMI-C SMI > SMI-C	.24	31.4****	HC > SMI,SMI-C SMI > SMI-C	.34
GFRS^f^	0.0 (1.0)	−0.2 (0.9)	−0.3 (0.9)	−0.4 (0.9)	−0.6 (0.9)	−1.0 (0.6)	4.3**	HC > SMI-C	.05	8.5****	HC,SMI > SMI-C	.12
*Chronotype*												
rMEQ	8.8 (2.0)	8.8 (2.1)	9.0 (1.6)	9.0 (2.1)	9.0 (1.4)	8.9 (1.7)	NS		NS			
rMEQ^g^	14 (24%)	6 (13%)	15 (25%)	9 (19%)	13 (28%)	8 (24%)	NS		NS			

*Note*: T1 ,time 1; T2, time 2; HC, healthy control; SMI, severe mental illness; C, comorbidity; HDRS, Hamilton rating scale for depression; YMRS, Young mania rating scale; PANSS, positive and negative syndrome scale; P, positive; N, negative; G, general; GCS, global cognitive score; GFS, global functional score; GFRS, global frailty score; rMEQ, reduced morningness-eveningness questionnaire; NS, not significant.

a Expressed as mean (standard deviation), except when indicated.

b MANCOVA.

c Bonferroni test.

d Partial eta-squared (*η* ^2^*p*).

e Lower scores represent a better outcome.

f *Z*-scores expressed as mean (standard deviation).

g Evening type *n* (%).

(NS = *p* > 0.05; *p ≤ 0.05; **p ≤ 0.01; ***p ≤ 0.001; ****p ≤ 0.0001). Effect size (*η* ^2^*p*: small ≈ 0.02; moderate ≈ 0.15; large ≈ 0.35).

Regarding neurocognition, compared to the HCs, both SMI groups had significantly lower GCS at both time points, being more accentuated in people with SMI-C (*p* < 0.0001; *η* ^2^*p* = 0.12–0.14). Similarly, the two groups with SMIs significantly underperformed HCs in GFS (*p* < 0.0001; *η* ^2^*p* = 0.24–0.34) at both times. It is important to note that individuals in the group with SMI-C demonstrated consistently lower neurocognitive and functional performance than those with SMI without comorbidities, indicating a clinically significant gradient associated with the presence of physical comorbidities. The magnitude of these differences was moderate (*η* ^2^*p* = 0.12–0.34), suggesting that the presence of comorbid physical conditions is associated with substantial reductions in overall cognitive and functional performance.

Moreover, individuals with SMI-C showed significantly lower GFRS than HCs at both times (*p* < 0.01–0.0001; *η* ^2^*p* = 0.05–0.12) and, also, for the SMI group at T2. In contrast, no differences in chronotypes were observed between the groups at either time point. Small-to-moderate effect sizes were observed for psychiatric symptoms and GCS, GFS, and GFRS scores in the between-group comparisons at both assessments. No significant within-group differences were observed over time for any of the studied variables.

Overall, these results suggest that, while chronotype preference was similar across all groups, individuals with severe mental disorders, particularly those with comorbid physical conditions, exhibited a significantly higher burden of psychiatric symptoms and poorer neurocognitive, functional, and frailty outcomes.

### Between-group comparison of hematological biomarkers

Peripheral biomarkers at T1 and T2 in the three groups are shown in Table 3. Overall, healthy controls (HCs) showed significantly lower values for hematological biomarkers at both time points. Among the erythrocyte indices, MCV proved to be the most reliable indicator for distinguishing between groups, with significantly higher values in the SMI-C group compared with healthy controls at both time points (*p* < 0.001–0.0001; *η* ^2^*p* = 0.10–0.11). Similarly, the SMI-C group exhibited significantly higher scores in MCH at T1 (*p* < 0.05; *η* ^2^*p* = 0.04). By contrast, MCHC was significantly higher in HCs compared to the SMI-C group at both times (*p* < 0.01; *η* ^2^*p* = 0.07). In all the cases, the effect sizes ranged from small to moderate. Within-group differences across time were not significant for any biomarker in any of the groups. These findings suggest subtle alterations in erythrocyte morphology in individuals with SMI and physical comorbidities.

**Table 3.** Red blood cell profile. Between-group comparison at Time 1 and Time 2

Variables^a^	HC		SMI		SMI-C		Statistical analyses					
	T1 (*n* = 58)	T2 (*n* = 44)	T1 *(n* = 60)	T2 (*n* = 48)	T1 (*n* = 47)	T2 (*n* = 33)	T1 F(*p*)^b^	Post hoc test^c^	*η* ^2^*p* ^d^	T2 F(*p*)^b^	Post hoc test^c^	*η* ^2^*p* ^d^
*Red blood cell count and oxygen transport*												
RBC	4.6 (0.4)	4.6 (0.4)	4.6 (0.3)	4.7 (0.3)	4.6 (0.4)	4.6 (0.3)	NS			NS		
HGB	13.7 (1.2)	13.5 (1.5)	13.9 (1.3)	13.9 (1.3)	13.8 (1.2)	13.8 (1.4)	NS			NS		
HCT	41.2 (3.6)	41.0 (4.2)	42.1 (4.0)	42.6 (3.2)	42.4 (3.9)	42.6 (4.1)	NS			NS		
*Erythrocyte morphological indices*												
MCV	88.4 (4.5)	87.4 (5.4)	89.8 (5.3)	90.8 (4.6)	93.0 (4.8)	90.9 (4.6)	9.4****	HC,SMI < SMI-C	.10	7.6***	HC < SMI,SMI-C	.11
MCH	29.3 (1.7)	29.1 (2.0)	29.7 (2.2)	29.6 (1.9)	30.3 (1.5)	29.6 (1.9)	3.8*	HC < SMI-C	.04	NS		
MCHC	33.3 (0.8)	33.2 (0.9)	33.0 (1.2)	32.7 (1.1)	32.5 (1.2)	32.5 (1.1)	5.9**	HC > SMI-C	.07	4.4**	HC > SMI-C	.07
RDW	13.6 (0.8)	13.7 (1.0)	13.7 (1.1)	13.7 (1.2)	13.7 (1.4)	14.1 (1.4)	NS			NS		

*Note*: T1, time 1; T2, time 2; HC, healthy control; SMI, severe mental illness; C, comorbidity; RBC, red blood cells; HGB, hemoglobin; HCT, hematocrit; MCV, mean corpuscular volume; MCH, mean corpuscular hemoglobin; MCHC, mean corpuscular hemoglobin concentration; RDW, red blood cell distribution width; NS, not significant.

a Expressed as mean (standard deviation), except when indicated.

b MANCOVA.

c Bonferroni test.

d Partial eta-squared (*η* ^2^*p*).

(NS = *p* > 0.05; *p ≤ 0.05; **p ≤ 0.01; ***p ≤ 0.001; ****p ≤ 0.0001). Effect size (*η* ^2^*p*: small ≈ 0.02; moderate ≈ 0.15; large ≈ 0.35).

### Predictive ability of psychiatric symptoms, neurocognition, functional performance, frailty, and hematological biomarkers at T1 of chronotype at T2

The distribution of chronotypes showed no significant differences between the groups at either assessment time point, indicating that circadian preference profiles are comparable among participants in the HC, SMI, and SMI-C groups. This finding provides the interpretive framework for subsequent predictive analyses.

The relative contributions of psychiatric symptoms, neurocognition, functional performance, frailty, and hematological biomarkers at T1 to the chronotype at T2 are shown in Table 4. In the HC group, the combination of depressive symptoms, GCS, GFS, and GFRS significantly predicted chronotype at T2, explaining 11.4% of the variance. Similarly, in the SMI group, the combination of maniac and positive psychotic symptoms together with GFRS significantly predicted the chronotype at T2 and explained 10.5% of the variance. In the SMI-C group, the combination of maniac mood, positive and negative psychotic symptoms, GFRS, and MCHC explained 23.9% of the chronotypic variance at T2. The predictive models explained a modest proportion of the variance in chronotype in the HC and SMI groups (10–11%), while the explanatory power was notably higher in the SMI-C group and frailty (23.9%), suggesting that the interaction between psychopathology, frailty, and hematological factors may be particularly relevant in the presence of comorbid physical illnesses. Across all groups, chronotype showed a positive correlation with GCS (*p* < 0.05), PANSS-P (*p* < 0.05–0.01), and MCHC (*p* < 0.05), and a negative correlation with HDRS (*p* < 0.05), YMRS (*p* < 0.05), PANSS-N (*p* < 0.05), GFS (*p* < 0.05), and GFRS (*p* < 0.05).

**Table 4.** Predictive psychiatric symptoms, neurocognition, functional performance, frailty, and hematological biomarkers at T1 of chronotype at T2

Dependent variables at T2	Predictors at T1 associated	*β*	95% CI	*t*	Percent of variance explained (adjusted *R* ^2^)
Group: HC					
ChrT	HDRS	−0.51	−3.14 to −0.06	2.1*	11.4
	GCS	0.31	−0.52 to 7.06	1.8*	
	GFS	−0.52	−11.16 to −0.45	2.1*	
	GFRS	−0.35	−7.45 to −0.12	2.0*	
Group: SMI					
ChrT	YMRS	−0.39	−1.89 to 0.18	1.8*	10.5
	PANSS-P	0.53	0.13 to 2.20	2.2*	
	GFRS	−0.20	−0.95 to 5.85	1.7*	
Group: SMI-C					
ChrT	YMRS	−0.32	−1.57 to 0.10	1.8*	23.9
	PANSS-P	0.81	1.21 to 4.00	3.8**	
	PANSS-N	−0.32	−0.88 to 0.03	1.9*	
	GFRS	−0.24	−5.46 to 0.81	1.7*	
	MCHC	0.34	0.01 to 5.44	2.0*	

*Note*: T1, time 1; T2, time 2; HC, healthy control; SMI, severe mental illness; C, comorbidity; ChrT, chronotype; HDRS, Hamilton rating scale for depression; YMRS, Young mania rating scale; PANSS, positive and negative syndrome scale; P, positive; N, negative; GCS, global cognitive score; GFS, global functional score; GFRS, global frailty score; MCHC, mean corpuscular hemoglobin concentration.

Significant level: *p ≤ 0.05; **p ≤ 0.01; ***p ≤ 0.001; ****p ≤ 0.0001.

### Discriminatory ability of psychiatric symptoms, neurocognition, functional performance, frailty, and hematological biomarkers at T1 to differentiate individuals with evening chronotype at 1-year follow-up

The ability of psychiatric symptoms, neurocognition, functional performance, frailty, and hematological biomarkers at T1 to differentiate individuals with evening chronotypes at 1-year follow-up is presented in Table 5. The combination of psychiatric symptoms (PANSS-P and PANSS-G) and hematological biomarkers (MCV, MCH, and MCHC) resulted in a model those best-discriminated individuals with evening chronotypes, with a correct differentiation rate of 83.1%. There was a positive correlation between the evening chronotype and PANSS-G (*p* < 0.05) and MCH (*p* < 0.05), and a negative correlation with PANSS-P (*p* < 0.05), MCV (*p* < 0.05), and MCHC (*p* < 0.05). The model demonstrated good discriminatory power (AUC = 0.84) and explained ~21% of the variance (Nagelkerke *R* ^2^ = 0.21). Taken together, these results suggest that an evening chronotype at follow-up is more closely associated with psychopathological severity and erythrocyte-related biomarkers than with neurocognitive or functional indicators alone.

**Table 5.** Psychiatric symptoms, neurocognition, functional performance, frailty, and hematological biomarkers at T1, with the ability to discriminate individuals with evening chronotype at 1-year follow-up

Dependent variables	Predictors at T1 associated	*β*	Wald’s test	Percent of variance explained	Global percentage correctly predicted
EC	PANSS-P	−0.28	4.1*	10–15%	83.1
	PANSS-G	0.07	4.7*		
	MCV	−0.44	4.7*		
	MCH	1.28	4.9*		
	MCHC	−0.95	3.6*		

*Note*: T1, time 1; EC, evening chronotype; PANSS, positive and negative syndrome scale; P, positive; G, general; MCV, mean corpuscular volume; MCH, mean corpuscular hemoglobin; MCHC, mean corpuscular hemoglobin concentration.

Significant level: *p ≤ 0.05; **p ≤ 0.01; ***p ≤ 0.001; ****p ≤ 0.0001.

## Discussion

To the best of our knowledge, this is the first longitudinal study to examine the clinical outcomes and peripheral hematological biomarkers associated with an evening chronotype in individuals with SMI, adopting a transdiagnostic perspective that also takes physical comorbidities into account.

**Figure 1.**
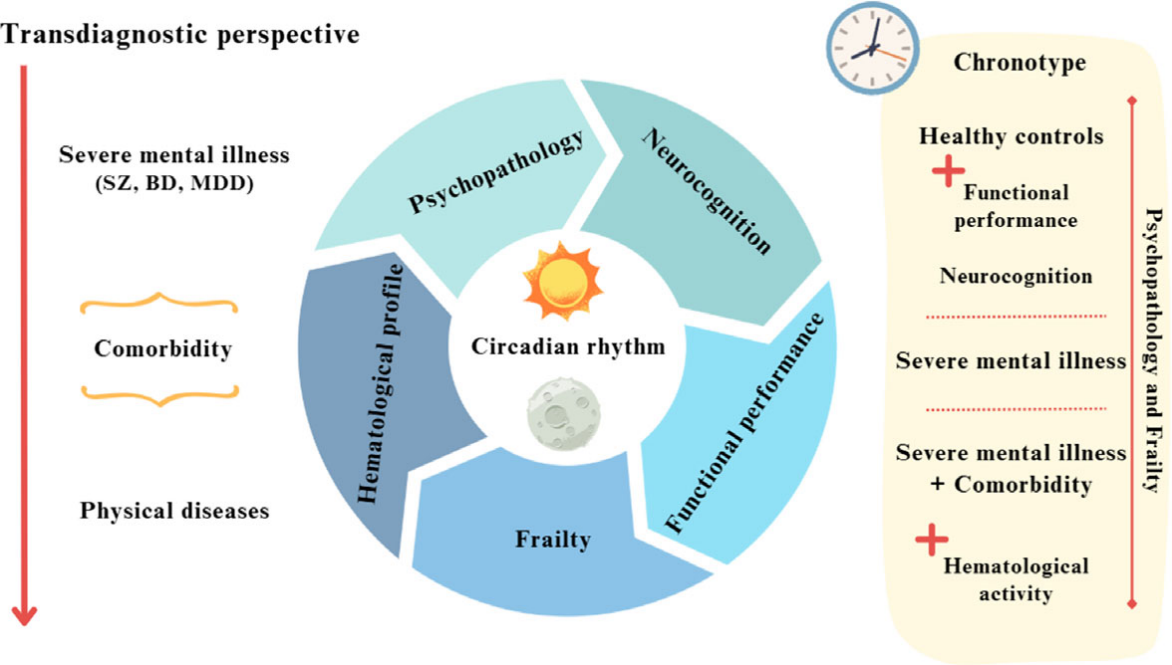
Summary of the main findings.

The main findings can be summarized as follows. First, individuals with SMI, especially those with physical comorbidities, exhibited greater depressive symptoms, poorer neurocognitive and functional performance, and higher levels of frailty. Second, alterations in erythrocyte indices (higher MCV and MCH and lower MCHC) were observed primarily in the SMI-C group. Third, the distribution of chronotypes was similar across all groups; however, longitudinal analyses indicated that psychopathology and frailty predicted chronotype over time. Finally, psychotic symptoms and erythrocyte-related biomarkers helped distinguish individuals with an evening chronotype at follow-up (see Figure 1).

Although chronotype has often been seen as a predictor of psychiatric and cardiometabolic outcomes, our results indicate that circadian preference may also be shaped by wider clinical and biological factors. From this perspective, chronotype might reflect the interplay between psychopathology, frailty, and systemic physiological processes, rather than functioning solely as a stable antecedent trait.

Overall, our findings are consistent with the chronotypic findings of SMIs (Zou et al., 2022). Taken together, these results indicate that, although the distribution of chronotypes did not vary across groups, vulnerability and psychopathological severity emerged as longitudinal predictors of chronotype in populations with severe mental disorders. Furthermore, erythrocyte-related biomarkers helped distinguish individuals with an evening chronotype, suggesting that circadian preferences may reflect broader clinical and physiological states.

These findings build upon growing evidence suggesting that physical comorbidities, regardless of the SMI diagnosis, similarly contribute to neurocognitive and functional impairments. Recent studies indicate that individuals with psychosis, particularly those with schizophrenia (SZ) and cardiometabolic comorbidities, show increased rates of neurocognitive and functional decline (Hagi et al., 2021). A similar scenario has been described in people with mood disorders (Arnaud et al., 2022; Kang et al., 2024). These findings are consistent with previous studies that point to a strong link between physical comorbidities, increased psychopathology, and greater frailty. For example, increased psychopathology, especially depressive symptoms, is one of the main risk factors for the onset of cardiovascular disease, and alterations in the levels of metabolic components are risk factors for depressive symptoms (Hebl et al., 2022; Kim, Wolf, & Kim, 2023; Tsapakis et al., 2024). Conversely, frail individuals have an increased risk of developing comorbidities, particularly when SMI is the index condition (Pearson et al., 2022). In summary, accumulating evidence seems to support the proposition that conditions, SMI, and physical comorbidities potentiate each other, and their combination negatively impacts clinical outcomes (Berk et al., 2023; Correll et al., 2022; Halstead et al., 2024).

Concurrently, several pathophysiological pathways, such as hematological processes, involved in the progression of physical diseases, may also contribute to the development of comorbidities in individuals with SMI (Krupa, 2025; Sánchez-Valle et al., 2020; Teixeira et al., 2022). Our results are consistent with previous findings, suggesting that hemoglobin (HGB) levels are elevated in individuals with schizophrenia spectrum disorders (Tang et al., 2022) and mood disorders (Beran et al., 2022; Tan et al., 2023). Similar results have also been found in populations with SMIs and cardiometabolic comorbidities, such as obesity or diabetes, reporting higher HGB levels when both diseases were present (Holsen et al., 2021; Pillinger et al., 2023). Similarly, altered RBC levels have been associated with the severity of psychopathology (e.g. negative and depressive symptoms) (Kriegmair et al., 2023; Rodrigues et al., 2024). To our knowledge, no previous studies have investigated these associations in a combined transdiagnostic sample, highlighting the relevance of this as an emerging area of research. Although the longitudinal design provides preliminary evidence that initial clinical characteristics may influence chronotype at follow-up, it is not possible to establish causal relationships. Therefore, the hematological abnormalities observed in this study may represent systemic physiological markers associated with health status, rather than direct causal mechanisms underlying differences in chronotype.

Regarding the results related to chronotypes, all groups showed similar patterns, consistent with previous findings in the literature (Boiko et al., 2024; Dollish, Tsyglakova, & McClung, 2024). Although chronotype profiles cannot yet be clearly established in individuals with SMIs, existing evidence suggests they play an important role in SMI pathophysiology (Linke & Jankowski, 2021). Although frailty and chronotype are connected, current research, including Deng et al.’s study, does not establish causality. Our longitudinal findings indicate that initial frailty could be associated with an evening chronotype at follow-up, but causation cannot be concluded (Deng et al., 2024). According to our findings, psychopathological features, such as mood and psychotic symptoms, may influence the occurrence of the evening chronotype in individuals with SMIs (Orsolini et al., 2023; Taillard, Sagaspe, Philip, & Bioulac, 2021), as do certain inflammatory and metabolic biomarkers in people with physical comorbidities (Baldanzi et al., 2022). Furthermore, the association between poor health status (e.g. depressive symptoms, frailty, and neurocognitive impairment) may increase the risk of evening chronotypes in HCs (Lotti et al., 2022). Therefore, from a transdiagnostic and comorbidity perspective, our findings provide evidence that overlapping psychopathology and frailty may contribute to the emergence of evening chronotypes and circadian rhythm disruption in SMIs.

Our findings build on growing evidence suggesting that individuals with SMIs share several pathogenic pathways, including alterations in hematological mechanisms (McQuaid, 2021). The complex relationship between psychopathology and hematological processes may influence the development of comorbidities (Teixeira et al., 2022). For instance, recent evidence has shown that differences in hematological profiles (e.g. mean platelet volume and RDW) can help identify depression and anxiety symptoms in people with autoimmune disorders (Fábián, Horváth, Shemirani, & Csiki, 2022), as well as differentiate individuals with depression from healthy controls with or without physical comorbidities, especially cardiometabolic diseases (Lin et al., 2021). Our results also suggest that psychopathology, in combination with hematological activity, plays a role in distinguishing individuals with an evening chronotype over time and converges with those of recent studies. In this regard, an impaired circadian rhythm can increase the vulnerability to neurodegenerative disorders (Liu et al., 2023) and has been postulated to be a potential predictor of poorer clinical outcomes over time in mood disorders (McCarthy et al., 2022). It also remains to be determined whether shared hematological mechanisms between SMIs and physical comorbidities may help define chronotype profiles in this population (Zou et al., 2022).

However, from a clinical perspective, the specific contribution of chronotype, frailty, neurocognitive and functional impairments, and hematological activity to the comorbidity profile in SMI remains unclear. The possibility of investigating subsets of individuals with specific chronotypes remains to be explored therapeutically, as these individuals might respond better than others to specific strategies (e.g. lifestyle-based interventions or anti-inflammatory treatment). Possible chronobiology-based strategies include interventions aimed at stabilizing circadian rhythms, such as light therapy, regulating sleep and wake schedules, structured physical activity programs, and behavioral approaches that promote consistent daily routines. In addition, interventions targeting inflammatory or metabolic dysregulation may indirectly influence circadian functioning.

It should also be noted that increases in erythrocyte indices, such as MCV or MCH, may be influenced by factors that were not assessed in this study. Nutritional status (e.g. vitamin B12 or folate levels), alcohol consumption, and pharmacological treatments commonly used in the psychiatric population, such as antipsychotics or mood stabilizers (e.g. valproate), may contribute to variations in these hematological parameters. Therefore, these potential influences must be taken into account when interpreting the biological significance of the results.

This study has several limitations. First, although the Charlson Comorbidity Index (CCI) is widely used to assess comorbidity, it does not fully capture its complexity (Drosdowsky & Gough, 2022). It would have been preferable to use more reliable measures of comorbidities, such as electronic medical records. Second, we did not evaluate other parameters involved in comorbidity pathophysiology, such as metabolic disruptions, changes in inflammatory activity, and vascular dysfunction biomarkers. This limitation is particularly relevant, as metabolic and inflammatory processes are closely linked to both circadian regulation and the development of physical comorbidities in people with severe mental disorders. Future studies that incorporate metabolic, inflammatory, and hematological biomarkers could provide a more comprehensive understanding of the biological pathways involved. Third, hematological processes among participants could fluctuate depending on the type and clinical status of the disorder and pharmacological treatments (Tatay-Manteiga et al., 2018). Furthermore, the potential role of lifestyle behaviors on chronotypes (Taillard et al., 2021) was not considered in the present analysis. Fourth, several relevant variables, such as current smoking status, were not collected at baseline. It should be noted that the PANSS negative symptoms subscale may not fully capture all aspects of negative symptoms, which could limit the interpretation of its contribution to chronotype. Fifth, high sample attrition was observed after a year of follow-up, and the follow-up period was short. Moreover, the HC group was selected as a convenience sample, which may limit the generalizability of the results. This may have introduced bias, favoring the retention of individuals in better clinical conditions. Sixth, the relatively small sample size reduced the generalizability of the results to populations with clinical characteristics similar to those of other studies. Despite these limitations, this study included a novel transdiagnostic and comorbidity approach, together with a comprehensive assessment of hematological biomarkers and neurocognitive and functional performance across individuals with SMIs. Moreover, the longitudinal design allowed the establishment of potential causal relationships between the included variables (e.g. hematological profile, neurocognitive performance, frailty, and chronotype). Finally, the multicenter nature of the study increases the external validity of the results.

In conclusion, our findings highlight that individuals with SMIs and comorbid physical conditions are at increased risk for neurocognitive and functional impairments. It is worth noting that this study provides one of the first longitudinal transdiagnostic analyses to integrate frailty, hematological biomarkers, and chronotype in individuals with SMI. Frailty appears to be a defining feature in this population. Multiple factors may contribute to these outcomes, including circadian rhythm disruptions, shared pathophysiological mechanisms (e.g. altered red blood cell indices), and bidirectional interactions between psychiatric and somatic health. Our results suggest a possible link between increased psychopathological burden and greater frailty in individuals with severe mental disorders, which may contribute to variability in chronotype.

Specifically, maintaining a healthy hematological system and stable psychopathology may be essential for discerning evening chronotypes in this population. These data provide new clinical approaches for addressing the chronotypes of individuals with SMIs. Managing the hematologic profile and identifying neurocognitive and functional impairments, as well as frailty, may help regulate circadian rhythm patterns in these disorders.

## Supporting information

10.1017/S0033291726104425.sm001 Sánchez-Ortí et al. supplementary material Sánchez-Ortí et al. supplementary material

## Data Availability

Data will be available upon request.

## Supplementary material

The supplementary material for this article can be found at http://doi.org/10.1017/S0033291726104425.
