# Supplementary material for: Commonalities in frailty and psychopathology predict chronotype across severe mental disorders from a comorbidity perspective
Source: Psychol Med. 2026 May 12;56:e143. doi: 10.1017/S0033291726104425 (PMC13200153; doi:10.1017/S0033291726104425)
Supplement: Sánchez-Ortí et al. supplementary material [file S0033291726104425sup001.docx]

– SUPPLEMENTARY MATERIAL –

COMORBIDITY

| **Supplementary material 1. Comorbidities** | | | | | | | | |
| --- | --- | --- | --- | --- | --- | --- | --- | --- |
|  | **MDD** | | BD | | **SZ** | | ***Statistical analyses*** | |
| **CCI^a^** | **T1**  (*n*=35) | **T2**  (*n*=25) | **T1**  *(n*=42) | **T2**  (*n*=29) | **T1**  (*n*=30) | **T2**  (*n*=27) | **T1**  **(*p*)^b^** | **T2**  **(*p*)^b^** |
| ***Number of comorbidity*** | | | | | | | | |
| **Hypertension** | 26(74.3%) | 15(60%) | 32(76.1%) | 20(68.9%) | 28(93.3%) | 24(88.8%) | NS | NS |
| **Heart disease** | 34(97.1%) | 24(96%) | 38(90.4%) | 25(86.2%) | 30(100%) | 27(100%) | NS | NS |
| **Cerebral disease** | 33(94.2%) | 24(96%) | 38(90.4%) | 26(89.6%) | 29(96.6%) | 25(92.5%) | NS | NS |
| **Respiratory disease** | 30(85.7%) | 21(84%) | 35(83.3%) | 26(89.6%) | 26(86.6%) | 22(81.4%) | NS | NS |
| **Renal disease** | 31(88.5%) | 23(92%) | 40(95.2%) | 26(89.6%) | 28(93.3%) | 27(100%) | NS | NS |
| **Liver disease** | 33(94.2%) | 23(92%) | 35(83.3%) | 27(93.1%) | 30(100%) | 27(100%) | NS | NS |
| **Gastrointestinal disease** | 32(91.4%) | 20(80%) | 38(90.4%) | 24(82.7%) | 28(93.3%) | 26(96.2%) | NS | NS |
| **Peripheral vascular disease** | 34(97.1%) | 24(96%) | 42(100%) | 29(100%) | 30(100%) | 27(100%) | NS | NS |
| **Malignancy** | 31(88.5%) | 21(84%) | 38(90.4%) | 23(79.3%) | 30(100%) | 27(100%) | NS | NS |
| **Locomotor impairment** | 25(71.4%) | 18(72%) | 31(73.8%) | 22(75.8%) | 29(96.6%) | 24(88.8%) | NS | NS |
| **Alcoholism** | 35(100%) | 25(100%) | 42(100%) | 29(100%) | 29(96.6%) | 26(96.2%) | NS | NS |
| **Miscellaneous** | 23(65.7%) | 24(96%) | 32(76.1%) | 20(68.9%) | 29(96.6%) | 25(92.5%) | NS | NS |
| **Myocardial infarction** | 35(100%) | 24(96%) | 42(100%) | 29(100%) | 30(100%) | 27(100%) | NS | NS |
| **Heart failure** | 35(100%) | 24(96%) | 38(90.4%) | 25(86.2%) | 30(100%) | 27(100%) | NS | NS |
| **Peripheral artery disease** | 35(100%) | 24(96%) | 42(100%) | 28(96.5%) | 30(100%) | 27(100%) | NS | NS |
| **Cerebrovascular disease** | 33(94.2%) | 24(96%) | 38(90.4%) | 26(89.6%) | 29(96.6%) | 26(96.2%) | NS | NS |
| **Dementia** | 35(100%) | 25(100%) | 42(100%) | 29(100%) | 30(100%) | 27(100%) | NS | NS |
| **Chronic respiratory disease** | 30(85.7%) | 22(88%) | 34(80.9%) | 26(89.6%) | 25(83.3%) | 24(88.8%) | NS | NS |
| **Connective tissue disease** | 31(88.5%) | 23(92%) | 38(90.4%) | 25(86.2%) | 30(100%) | 27(100%) | NS | NS |
| **Gastroduodenal ulcer** | 32(91.4%) | 20(80%) | 38(90.4%) | 24(82.7%) | 28(93.3%) | 26(96.2%) | NS | NS |
| **Mild chronic liver disease** | 33(94.2%) | 23(92%) | 35(83.3%) | 27(93.1%) | 30(100%) | 27(100%) | NS | NS |
| **Diabetes** | 27(77.1%) | 18(72%) | 31(73.8%) | 23(79.3%) | 26(86.6%) | 23(85.1%) | NS | NS |
| **Hemiplegia** | 35(100%) | 24(96%) | 42(100%) | 27(93.1%) | 29(96.6%) | 27(100%) | NS | NS |
| **Chronic renal failure** | 31(88.5%) | 23(92%) | 42(100%) | 26(89.6%) | 29(96.6%) | 27(100%) | NS | NS |
| **Diabetes with target organ damage** | 35(100%) | 25(100%) | 42(100%) | 29(100%) | 30(100%) | 26(96.2%) | NS | NS |
| **Solid neoplasm** | 32(91.4%) | 22(88%) | 38(90.4%) | 25(86.2%) | 30(100%) | 27(100%) | NS | NS |
| **Leukemia** | 35(100%) | 25(100%) | 42(100%) | 29(100%) | 30(100%) | 27(100%) | NS | NS |
| **Lymphoma** | 34(97.1%) | 24(96%) | 42(100%) | 29(100%) | 30(100%) | 27(100%) | NS | NS |
| **Chronic liver disease** | 35(100%) | 25(100%) | 42(100%) | 29(100%) | 30(100%) | 27(100%) | NS | NS |
| **Solid neoplasm with metastasis** | 35(100%) | 25(100%) | 42(100%) | 27(93.1%) | 30(100%) | 27(100%) | NS | NS |
| **Definite AIDS** | 35(100%) | 25(100%) | 42(100%) | 29(100%) | 30(100%) | 27(100%) | NS | NS |
| ***Comorbid size*** | | | | | | | | |
| **Severity of disease** | 1.1(1.5) | 1.4(1.9) | 1.0(1.4) | 1.3(1.2) | 0.5(0.9) | 0.4(0.6) | NS | NS |
| ^a^ Expressed as absence of disease n(%), ^b^ Chi-squared test. ^c^ ANOVA. Abbreviations: T1 = time 1, CCI = Charlson comorbidity index, HC = healthy control, SMI = severe mental illness, C = comorbidity, AIDS = Acquired immune deficiency syndrome. | | | | | | | | |


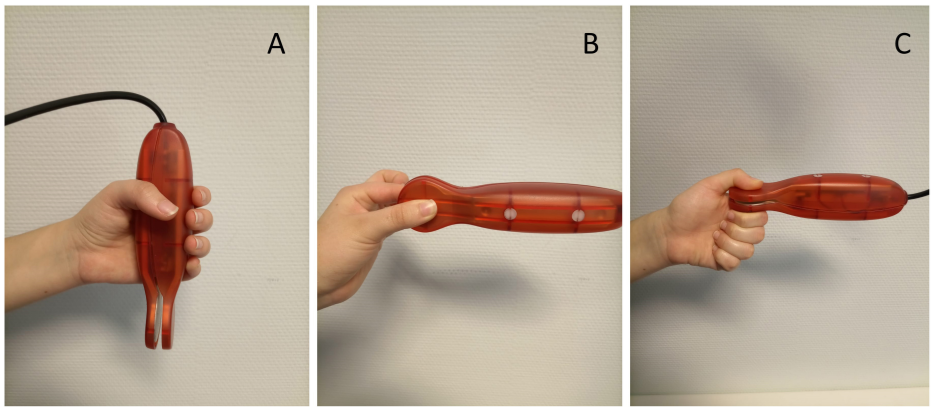


**Supplementary material 2. Electronic dynamometer (NedVEP/IBV) functional positions: (A) handgrip, (B) lateral/key pinch (thumb pad and lateral aspect of index finger), (C) tip**

**pinch (thumb opposed by the index and long fingers)**
